# Human adenovirus type 7 subunit vaccine induces dendritic cell maturation through the TLR4/NF-κB pathway is highly immunogenic

**DOI:** 10.3389/fcimb.2023.1117230

**Published:** 2023-04-12

**Authors:** Yaru Li, Xia Yang, Renshuang Zhao, Zhiru Xiu, Shanzhi Li, Yue Li, Gaojie Song, Chenchen Ge, Jinbo Fang, Jicheng Han, Yilong Zhu, Yiquan Li

**Affiliations:** ^1^ Academician Workstation of Jilin Province, Changchun University of Chinese Medicine, Changchun, China; ^2^ Medical College, Yanbian University, Yanji, China; ^3^ Medical College, Jiujiang University, Jiujiang, China

**Keywords:** hexon, neutralizing antibody, dendritic cell, subunit vaccine, lymphocytes

## Abstract

**Introduction:**

Human adenovirus type 7 (HAdv-7) infection is the main cause of upper respiratory tract infection, bronchitis and pneumonia in children. At present, there are no anti- adenovirus drugs or preventive vaccines in the market. Therefore, it is necessary to develop a safe and effective anti-adenovirus type 7 vaccine.

**Methods:**

In this study, In this study, we used the baculovirus-insect cell expression system to design a recombinant subunit vaccine expressing adenovirus type 7 hexon protein (rBV-hexon) to induce high-level humoral and cellular immune responses. To evaluate the effectiveness of the vaccine, we first detected the expression of molecular markers on the surface of antigen presenting cells and the secretion of proinflammatory cytokines *in vitro*. We then measured the levels of neutralizing antibodies and T cell activation *in vivo*.

**Results:**

The results showed that the rBV-hexon recombinant subunit vaccine could promote DC maturation and improve its antigen uptake capability, including the TLR4/NF-κB pathway which upregulated the expression of MHCI, CD80, CD86 and cytokines. The vaccine also triggered a strong neutralizing antibody and cellular immune response, and activated T lymphocytes.

**Discussion:**

Therefore, the recombinant subunit vaccine rBV-hexon promoted promotes humoral and cellular immune responses, thereby has the potential to become a vaccine against HAdv-7.

## 1 Introduction

Human adenovirus (HAdv) infects humans and causes a variety of respiratory and intestinal diseases. In children, it is highly infectious and infection with HAdv often leads to diseases such as acute respiratory disease (ARD), which includes upper respiratory tract infection, bronchitis and pneumonia (Tsou et al., 2012; Foong Ng et al., 2015). In immunocompetent adults, symptoms resulting from an adenovirus infection are usually mild and self-limiting, but they can lead to serious consequences and even fatalities in children and individuals with low immune function (Tang et al., 2013; Cui et al., 2015; Zhang et al., 2016).

Adenoviruses are divided into seven subgroups: A, B, C, D, E, F and G. The most common types associated with respiratory infection are types 3, 7, 14 and 21 (subgroup B), types 1, 2 and 5 (subgroup C) and type 4 (subgroup E) (Carr et al., 2011; Wo et al., 2015). Among them, type B1 adenoviruses, HAdV-3, HAdV-7 and HAdV-55, are the main pathogens that cause adenovirus outbreaks in most regions of North America, Asia and Europe (Hai le et al., 2016; Bautista-Gogel et al., 2020; Li et al., 2021; Liu et al., 2022). Epidemiological reports have shown that the most deadly adenovirus infection in children may be related to human adenovirus type 7 (HAdV-7) (Cheneau and Kremer, 2020). However, little is known about the pathogenesis of severe diseases induced by HAdV-7 is still poorly understood. At present, there is also no anti-adenovirus drug available. there are no available anti-adenovirus drugs.

The administration of vaccines is an effective way to prevent infections. At present, there are no adenovirus vaccines available for public use, and only vaccines targeting HAdV types 4 and 7 have been developed for the US military (Russell et al., 2006). Therefore, in order to reduce the harmful consequences of an adenovirus type 7 infection in humans, it is necessary to develop a safe and effective anti-adenovirus type 7 vaccine. Studies have shown that specific neutralizing antibodies present in the serum are associated with the successful elimination of AdV (Heemskerk et al., 2005; Echavarria, 2008). Adenovirus capsid is composed of three major proteins (hexon, penton base and fiber) and four minor proteins (IIIa, VI, VIII and IX). The hexon protein is the standard for diagnosing different serotypes, including the antigen components of mammalian adenovirus. Furthermore, hexon protein is the target of neutralizing antigen, and its outer end contains two loop structures,. The loop structure is the binding site of where adenovirus and binds to serum antibody,. Loop 1 is the region of where HVR1-6 is located, and loop2 is the region of where HVR7 is located. Hexon contains blood group-specific B cell epitopes, so it is used for HAdV serotype typing. Hexon mutation can change the antigenicity of adenovirus, and these antigenicity changes can make adenovirus escape immunity (Yu et al., 2013; Su et al., 2016). Therefore, the use of the antigenic hexon protein is a good strategy for the development of an anti-adenovirus vaccine.

Compared with traditional inactivated vaccine and attenuated vaccine, genetically engineered subunit vaccine has higher antigen content and purity, and has many advantages such as strong specificity, non-infectivity, low cost, less restrictions, etc., and is the best choice for the research and development of vaccines for severe infectious diseases (Khalaj-Hedayati et al., 2020; Awadasseid et al., 2021). At present, subunit vaccines can be produced on a large scale by using Escherichia coli, baculovirus and Pichia pastoris as vectors. Among them, the baculovirus-insect cell expression system has the characteristics of easy operation, large capacity of accommodating foreign genes, high expression efficiency, high safety and low cost, and can modify the target protein after expression. In 1983, Pennock et al. successfully expressed Escherichia coli β –galactosidase by using insect cells, which marked the birth of baculovirus-insect cell expression system(Pennock et al., 1984). In recent years, the expression system has also been used in many virulent infectious disease vaccines, such as Covid-19 vaccine and influenza virus vaccine (Ruhnau et al., 2021; Yang et al., 2022; Pavot et al., 2023). The expression system has also been widely used in the field of animal vaccines. The vaccine developed using the baculovirus-insect cell expression system after fusion of the bluetongue virus VP2 gene with antigen-presenting cell homing molecule (APCH) has been found to enhance cellular immune response and VP2-induced neutralizing activity (Legisa et al., 2015). Other studies have shown that avian influenza H5 vaccine based on baculovirus-insect cell expression system, combined with inactivated Newcastle disease vaccine, has a significant clinical protection effect on highly pathogenic H5N1 and Newcastle disease virus (Said et al., 2019). Because baculovirus can only proliferate in insect cells, it is expected to use this characteristic to design a new virus vector vaccine to improve the safety of the current virus vector vaccine.

In this study, we selected the strategy of baculovirus vector expressing adenovirus type 7 hexon protein to construct the recombinant Baculovirus hexon (rBV-hexon) subunit vaccine. The vaccine construct can stably express the hexamer protein, which can not only promote DC maturation, but also effectively stimulate humoral and cellular immune responses, and does not lead to any dire effects in the vaccinated mice. This study provides an experimental basis for the future development of a safe and efficient recombinant subunit vaccine for human adenovirus type 7.

## 2 Materials and methods

### 2.1 Virus and animals

Human adenovirus type 7 was isolated and preserved in our laboratory. Female BALB/c mice aged 5 weeks old were purchased from the Experimental Animal Center of the Chinese Academy of Agricultural Sciences. The Institutional Animal Care and Use Committee (IACUC) of the Changchun University of Chinese Medicine approved the animal experimental protocols (Approval No. 2021079).

### 2.2 Construction of the subunit vaccine

The *hAd-7* hexon sequence was obtained from the Human adenovirus 7 strain BJ/CHN/2018 (GenBank: MH355567.1). The hexon gene was inserted into pFastBac HT A vector to obtain pFastBac-hexon recombinant plasmid. The recombinant plasmid was transformed into competent cells of DH10Bac™, coated on a blue-and-white spot screening plate containing tetracycline (10 mg/L), kanamycin sulfate (50 mg/L), gentamicin (7 mg/L) and IPTG/X-gal, and inverted at 37°C for 48 hours. White colonies were selected and added to SOC culture medium, and the recombinant baculocytes were extracted after shaking culture at 37°C for 3 hours. Then, 4 μg of recombinant baculovirus was mixed with 8 μL of Cellfectin II Reagent transfection reagent, and then added to 1.5 × 10^6^ viable Sf9 cells (Insect cell line)/ml. After standing at 27°C, the cell state was observed every day. When 60% of the cells showed CPE, the supernatant was collected as the first generation recombinant baculovirus, which was used to infect SF9 cells and spread to the third generation, and the recombinant baculovirus (rBV) containing the *hAd-7* hexon gene was obtained. To produce the recombinant subunit vaccine, 2.5 × 10^6^ viable Sf9 cells (Insect cell line)/ml were infected with rBV at an MOI of 5 and incubated at 27°C for 72 hours. The expressed protein was purified by sucrose gradient ultracentrifugation and analyzed by western blot.

### 2.3 Production of DC cells and uptake of DC antigen

The mouse primary bone marrow cells were isolated according to the methods described in a previous study (Han et al., 2021), and then cultured in SF-900 medium containing 10% Fetal Bovine Serum, 20 ng/ml GM-CSF and 20 ng/ml IL-4 for 6 days. DCs were then collected and subcultured into 6-well plates at 5 × 10^6^ cells per well. After 24h, 1 μg LPS (1μg/ml) and 10 μg rBV-hexon (5μg/ml) were added and the cells were further incubated for 48 hours. The DCs cells were then harvested and stained with FITC dextran (1mg/ml) at 37 °C and 4 °C for 2h respectively. Flow cytometry was used to analyze the difference in the mean fluorescence intensity (MFI). In the DC maturation experiment, supernatants obtained from the culture of antigen treated DCs were collected and used to detect the presence of IL-6 (R&D, cat.DY406-05), IL-12p70 (R&D, cat.M1270), TNFα (R&D, cat. DY410-05) and IFN-γ (R&D, cat. DY485-05) according to the instructions of the ELISA kit.

### 2.4 Mouse lymphocyte isolation

After euthanasia, the spleen was taken out under sterile conditions and smashed with a 70μm cell strainer to prepare a single-cell suspension. The spleen lymphocytes were extracted with Mouse Spleen Lymphocyte Separation Kit (Solarbio, cat. P8860), and the red blood cells contained in the splenic lymphocytes were removed with Red Blood Cell Lysis Buffer (Solarbio, cat. R1010).

### 2.5 Flow cytometry

DC (2 × 10^6^ cells) and spleen lymphocytes (2 × 10^6^ cells) were incubated with APC-CD11c (BioLegend, cat.117310), PE-CD80 (BioLegend, cat.104707), PE-CD86 (BioLegend, cat.105007), PE-CD40 (BioLegend, cat.124609), FITC-I-A/I-E (BioLegend, cat.), PE-CD3 (BioLegend, cat.100205), PerCP-CD4 (BioLegend, cat.100431), and FITC-CD8 (BioLegend, cat.100705) antibodies and the proportion of positive cells was analyzed by flow cytometry.

### 2.6 Inhibitor analysis

DCs (5 × 10^6^ cells) were pretreated with 100 nM TAK-242 (TLR4 inhibitor, MedChemExpress, USA) or 20 mM PDTC (NF-κB inhibitor, MedChemExpress, USA) for 2 hours, and then incubated with 10 μg rBV-hexon for 48 hours at 37 °C. Cell surface markers were detected by flow cytometry and cytokines were detected by the ELISA kit.

### 2.7 Animal immune assay

Five-week-old female BALB/c mice were randomly divided into 6 groups (n=6 per group) and immunized with the rBV protein at 0 and 21 days. Blood was collected weekly to detect the level of specific antibody and neutralizing antibody levels were detected according to the methods in previous study (Li et al., 2016). The mice were euthanized on the fifth week and spleens were collected for pathological analysis. Spleen lymphocytes were extracted with the Mouse Spleen Lymphocyte Separation Kit (Solarbio, cat. P8860) and the number of cells was cell numbers were adjusted to 1 × 10^6^/ml with RPMI1640 culture medium. The lymphocyte proliferation assay was performed with inactivated hAd-7 as the specific stimulator and Con A as the positive stimulator, as previously described (Han et al., 2021).

Extracted The extracted mouse spleen lymphocytes cells (2 × 10^6^ cells) were stained with anti-CD3, CD4, and CD8, and then detected by flow cytometry. The cChanges in CD3^+^CD4^+^ and CD3^+^CD8^+^ counts in each group of mice were determined. The cCells were then stained with anti-IL-4 and IFN-γ and flow cytometry was used to detect changes in CD3^+^CD4^+^/IL-4/IFN-γ and CD3^+^CD8^+^/IL-4/IFN-γ in each group of mice.

### 2.8 Clinical disease scores

Mice were observed daily for morphology, movement, respiration, and body weight changes throughout the experimental cycle, and a disease activity index (DAI) score was established (Blattner et al., 2016): (a) weight loss (0 point: no loss; 1-20 points: loss of 1-20%, 20 points: loss of over 20%; ); (b) Fur condition (0 point: shining; 2 points: matte; 4 points: ruffled); (c) Eyes condition (0 point: clear and clean; 3 points: unclean, closed); (d) Posture (0 point: normal; 10 points: hunched; 20 points: massively hunched); (e) Motility (0 point: normal; 1 point: spontaneous but reduced; 2 points: moderately reduced activity; 5 points: motility only after stimulation; 10 points: lethargy; 20 points: self-mutilation); (f) Respiratory condition (0 point: normal; 1 point: slightly changed; 10 points: accelerated breathing + 30% (tachypnoea); 20 points: strongly accelerated breathing + 50%); The daily DAI score is the sum of six items: A, B, C, D, E and F.

### 2.9 Histopathological scores

The histopathological scores of each tissue were as follows: extremely mild or no - 0 point, mild - 1 point, moderate - 2 points and severe - 3 points (**Table 1**).

**Table 1:** Scoring standards of various tissues.

Lung									
Alveolar atrophy collapse	Alveolar wall thickening			Cell degeneration		Inflammatory cell infiltration			bleed
Heart									
Myocardial cell degeneration			Inflammatory cell infiltration				bleed		
Liver									
Hepatocyte degeneration			Inflammatory cell infiltration				fibrosis		
Muscle									
Cell degeneration			Inflammatory cell infiltration				bleed		
Brain									
Neuronal degeneration			Glial cell hyperplasia				Tissue edema		
Kidney									
Renal tubular dilatation/degeneration		Cell degeneration			Glomerular atrophy or enlargement			Inflammatory cell infiltration	

### 2.10 Statistical analysis

All data are presented as the mean ± SD. We used GraphPadPrism 6.0 to perform statistical analysis or analysis of variance (ANOVA) of unpaired double-tailed Student’s test. *P* < 0.05 is considered statistically significant. *P < 0.05, **P < 0.01, ***P < 0.001.

## 3 Results

### 3.1 Expression and confirmation of rBV-hexon protein

**Figure 1:**
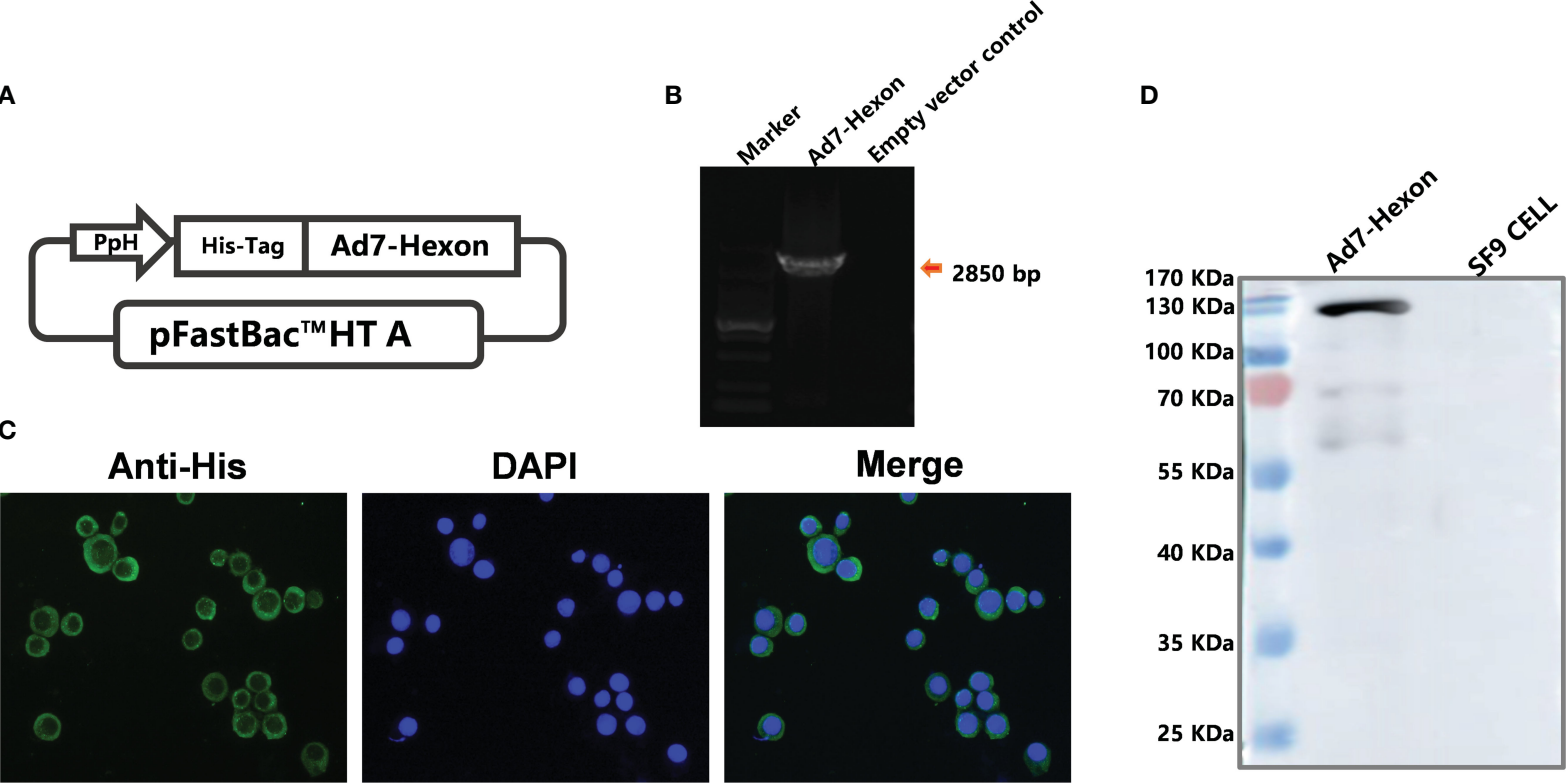
Construction of recombinant baculovirus carrying the *Ad7-hexon* gene and confirmation of the expressed adenovirus subunit vaccine type 7 rBV-hexon. **(A)** The adenovirus type 7 hexon gene was inserted into the pFastBac™ HT A vector. **(B)** Insertion of the *Adv7-hexon* gene into the recombinant baculovirus was confirmed by PCR. **(C)** An indirect immunofluorescence assay was performed to detect the expression of hexon protein using His-labeled antibody. **(D)** The adenovirus type 7 hexon protein was detected by western blot. The arrow indicates a destination stripe of 2850bp size.

Using the insect expression system, we constructed a recombinant baculovirus rBV expressing adenovirus type 7 hexon protein (**Figure 1A**) and PCR amplification confirmed the successful insertion of the hexon gene (2850 bp) (**Figure 1B**). Subsequently, the recombinant protein rBV-hexon was obtained by infecting SF-9 cells with rBV and subjected to western blot and indirect immunofluorescence experiments with His-labeled antibody. Successful expression of the hexon protein was observed (**Figures 1C, D**).

### 3.2 Antigen uptake on rBV-hexon-treated DCs

**Figure 2:**
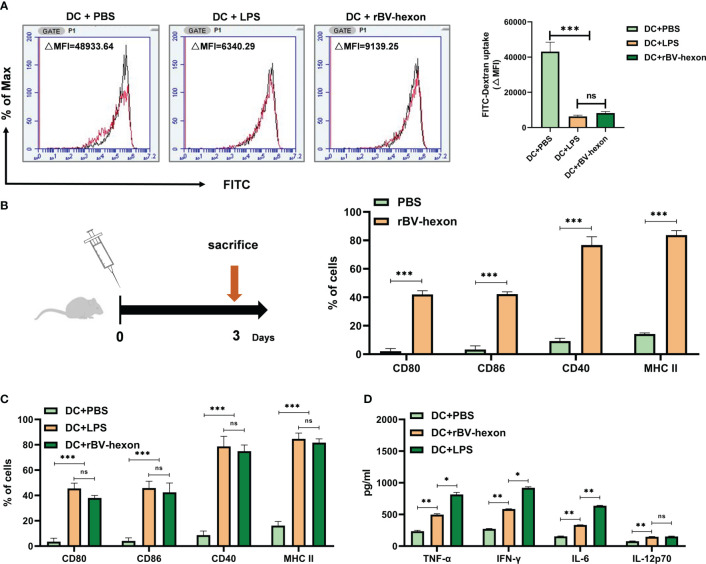
DCs can be induced to absorb antigen and mature by recombinant subunit vaccine rBV-hexon. **(A)** Fluorescence intensity of DCs incubated with PBS, LPS and rBV-hexon at 37°C was analyzed by flow cytometry. The samples at 4°C served as the negative control (Red). **(B)** Naïve mice (C57BL/6, n=6) were immunized with rBV-hexon and PBS was used as a negative control. After 3 days of immunization, spleen lymphocytes were isolated, and the percentages of CD80, CD86, CD40 and MHC-II expression in Dendritic cells were analyzed by flow cytometry. **(C)** BMDCs were incubated with PBS, LPS and rBV-hexon and the expression of cell surface markers was detected by flow cytometry. **(D)** Cytokine secretion was detected by ELISA after incubating BMDCs with PBS, lipopolysaccharide and rBV-hexon. The experiments were repeated with three independent experiments. Error bars reflect the standard deviation (SD). *P < 0.05; **P < 0.01, ***P < 0.001. The arrow indicates that the mice were sacrificed on the third day. ns, no significance.

BMDCs were incubated with PBS, LPS or rBV-hexon and stained with FITC-Dextran for analysis of DC antigen uptake. The results showed that DCs cells in the PBS treatment group (△MFI=48933.64) had the highest antigen uptake, while DCs cells in the rBV-hexon-treated group had significantly reduced antigen uptake (△MFI=9139.25) (*P <*0.001), which was similar to that in the LPS group (△MFI=6340.29) (**Figure 2A**). Maturation begins when DCs cease taking up antigens, hence, the data suggests that rBV-hexon can could induce dendritic cell maturation.

3. The vaccine can promote DC maturation and induce secretion of pro-inflammatory cytokines

DCs need the assistance of MHC II and the co-stimulatory molecules CD80 and CD86 to participate in the antigen presentation process. In the previous section, we demonstrated that rBV-hexon could induce dendritic cell maturation. To further verify if rBV-hexon affects the expression of DC surface molecules, we first immunized the mice with rBV-hexon for three days and then analyzed the expression levels of CD80, CD86, CD40 and MHCII in CD11c+ spleen lymphocytes by flow cytometry (**Figure 2B**). After immunization with rBV-hexon, Compared with PBS group, the expression of CD80, CD86, CD40, and MHCII was significantly increased significantly after rBV-hexon immunization. compared to the PBS group. The results indicate that rBV-hexon promotes antigen presentation *in vivo*.

Subsequently, we analyzed the effect of rBV-hexon on the expression of molecules on DCs surface *in vitro*. After incubating BMDCs with PBS, LPS and rBV-hexon, we analyzed the expression of MHC II, CD80, CD86 and CD40.We noted that DCs in the rBV-hexon group showed significant surface marker expression similar to that in the LPS group (**Figure 2C**). In addition, DC antigen presentation is known to release large quantities of pro-inflammatory cytokines. Therefore, we analyzed the release of TNFα, IFNγ, IL-6, and IL-12p70, and found that rBV-hexon significantly increased the secretion of these four cytokines (**Figure 2D**). These results indicated that rBV-hexon could activate DC maturation and induce the release of a large number of pro-inflammatory cytokines, which in turn could promote the proliferation and differentiation of other immune cells.

### 3.3 Inhibition of TLR4 and NF-κB decreases rBV-hexon-treated DC surface markers and proinflammatory cytokine levels

**Figure 3:**
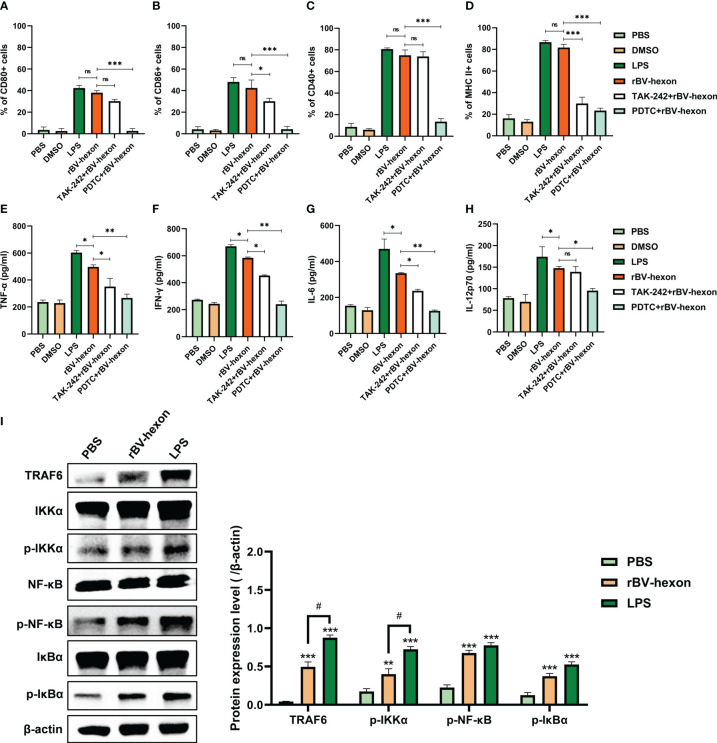
Blocking TLR4 and NF-κB inhibits DC maturation. After treatment with TLR4 and NF-kB inhibitors, DC surface molecules CD80 **(A)**, CD86 **(B)**, CD40 **(C)** and MHC II **(D)** were detected by flow cytometry. ELISA was used to test the secretion of TNFα **(E)**, IFNγ **(F)**, IL-6 **(G)** and IL-12p70 **(H)**. **(I)** Whole cell proteins were extracted, and the expression of key proteins in the TLR4/NF-κB pathway in macrophages was detected by Western blot. The experiments were repeated over three independent experiments. Error bars represent the standard deviation (SD). *P < 0.05; **P < 0.01, ***P < 0.001. P < 0.05. ns, no significance.

Studies have shown that the LPS receptor (TLR4) can recognize viral glycoprotein (Zhang et al., 2003), and that LPS-induced cytokines are mainly associated with NF-κB downstream of the TRL4 pathway (Kawai and Akira, 2011). In this study, we found that the expression levels of rBV-hexon-activated DCs surface markers were similar to those of LPS-activated DCs. Therefore, we hypothesized that rBV-hexon might activate DCs *via* the TLR4/NFκB pathway. In this study, we used inhibitors to inhibit TLR4 and NF-κB and established that the expression level of DCs surface markers was significantly decreased after PDTC (NF-κB inhibitor) treatment, while only MHC II and CD86 positive cells were significantly decreased after TAK242 (TLR4 inhibitor) treatment (**Figures 3A–D**). In terms of cytokine production (**Figures 3E–H**), both inhibitors inhibited the secretion of TNFα, IFNγ and IL-6, while TAK242 had a minor effect on IL-12p70. Then we analyzed the expression of TRAF6, IKKα/β, IκBα and NF-κB proteins (**Figure 3I**). The result shows that rBV-hexon can activate TRAF6 downstream of TLR4, and then activate IKK. The phosphorylation of IK will activate NF-κB signal transduction pathway. In conclusion, rBV-hexon activates DCs maturation *via* the TLR4/NF-κB pathway.

### 3.4 The vaccine can improve specific antibodies and neutralizing antibodies

**Figure 4:**
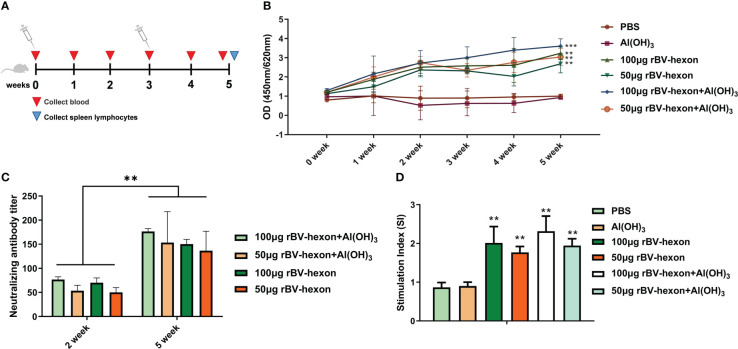
rBV-hexon increases specific and neutralizing antibody levels *in vivo*. **(A)** Mice (Balb/c, n=6) were immunized with 50μg rBV-hexon+Al(OH)_3_, 100μg rBV-hexon+Al(OH)_3_, 50μg rBV-hexon or 100μg rBV-hexon, and PBS was used as the negative control. **(B)** Blood samples were collected weekly and specific antibody titers were measured. **(C)** Detection of neutralizing antibody titers in the second week after primary and booster immunizations. **(D)** Lymphocyte proliferation was analyzed after the isolation of mouse spleen lymphocytes. The experiments were repeated over three independent experiments. Error bars represent standard deviation (SD). **P < 0.01, ***P < 0.001.

Weekly tail vein blood collection and testing for specific antibodies towards adenovirus type 7 were performed over the mice immunization period (**Figure 4A**). During the immune period of mice, blood was collected from the tail vein every week and specific antibodies against adenovirus type 7 were tested (**Figure 4A**). The results showed that the mouse specific antibody titers of mice in the vaccine group was were significantly higher (*P <*0.05) than that in those of the control group and the adjuvant group. From the second week onwards, the effect of the high dose vaccine group was superior to that of the low dose vaccine group, and the adjuvant (Al(OH)_3_) was able to improve the specific antibody levels induced by the vaccine (**Figure 4B**). We then tested for neutralizing antibodies at two weeks after the initial vaccine injection and found that the titer of neutralizing antibodies was 1:50 after the first injection and improved to 1:150 after the second immunization. The adjuvant was able to increase the level of the neutralizing antibodies produced by the vaccine (**Figure 4C**). The above results showed that the vaccine was able to produce high levels of specific and neutralizing antibodies, demonstrating a high level of immunogenicity.

### 3.5 Vaccine promotes the activation of T lymphocytes

Spleen lymphocytes were isolated from immunized mice and stimulated to induce lymphocyte proliferation. Compared to the adjuvant group and control group, when treated with the adenovirus type 7 antigen, the vaccine group could significantly stimulate the specific proliferation of lymphocytes (*P*<0.01), but there was no significant difference between the adjuvant and control groups (*P*>0.05) (**Figure 4D**).

**Figure 5:**
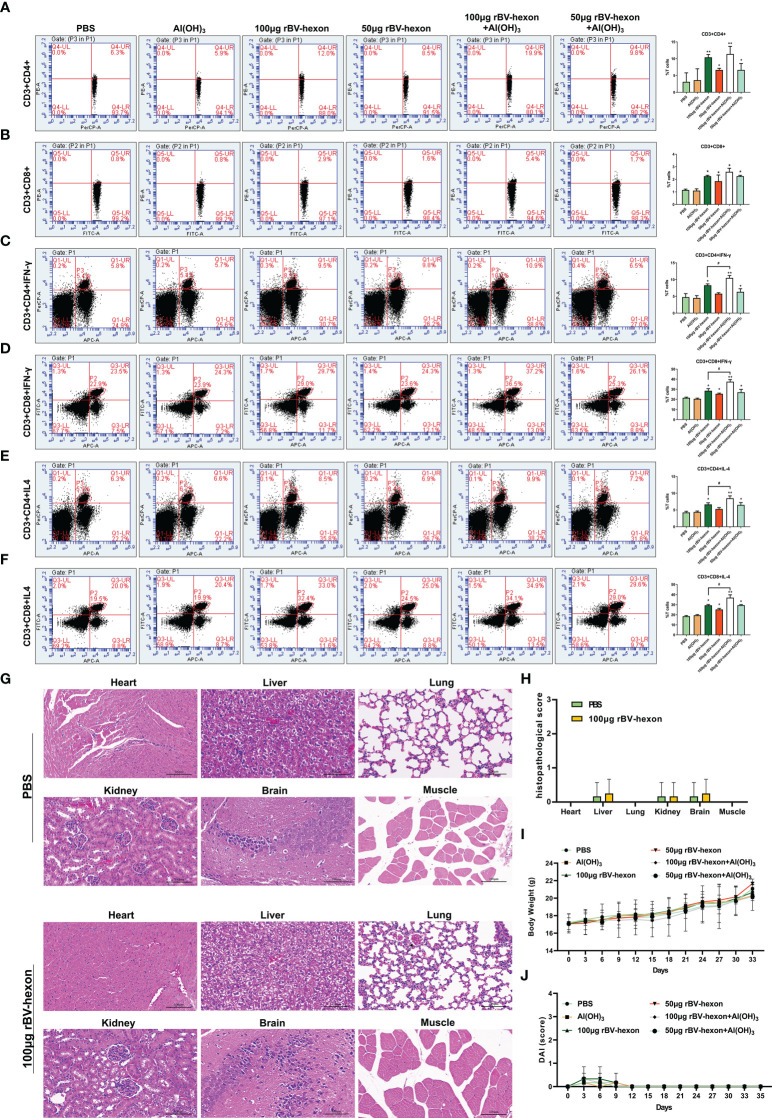
T-cell response induced by rBV-hexon inoculation. **(A, B)** Percentage of CD3^+^CD4^+^ and CD3^+^CD8^+^ cells in spleen lymphocytes detected by flow cytometry. **(C–F)** CD4^+^ and CD8^+^ T-cell activation was evaluated by detecting secretion of IFN-γ and IL-4. **(G)** HE staining was used to analyze the pathological changes of various organs and tissues. **(H)** Histopathological scores of mice. **(I)** Weight change of mice. **(J)** DAI score of mice. The experiments were repeated over three independent experiments. Error bars represent standard deviation (SD). *P < 0.05; **P < 0.01; #P < 0.05.

Lymphocyte typing showed that the proportion of CD4 and CD8 positive cells in the vaccine group was significantly higher (*P <*0.05) than that of the control group (**Figures 5A, B**; **S1**). Furthermore, the levels of CD4^+^IFN-γ and CD4^+^IL-4, CD8^+^IFN-γ and CD8^+^IL-4 in the vaccine group were higher than those in the control group (**Figures 5C–F**; **S2-3**). We also noted that the effectiveness of the vaccine improved in the presence of adjuvant and taken together, these results show that the vaccine is highly immunogenic *in vivo*. Pathological examination of important organs and muscles at the injection site of the immunized mice revealed no significant pathological changes in the tissues of immunized mice compared to the control group (**Figures 5G, H**). In addition, we can also find from the DAI score of mice that the immunized vaccine did not cause abnormal clinical symptoms in mice (**Figures 5I, J**). These findings imply that the rBV-hexon vaccine is safe for administration.

## 4 Discussion

Human adenovirus type 7 is an unencapsulated, icosahedral, double-stranded DNA virus. Because of its complex pathogenic mechanism and limited knowledge of its genotype diversity and protective antigens, there are no effective drugs and vaccines to prevent and control respiratory tract infections caused by this virus. Therefore, it is imperative to develop a safe and effective vaccine against the virus as soon as possible. At present, the adenovirus type 7 vaccine is only available for military personnel and not for the public. At present, the fastest way to develop a vaccine is to prepare inactivated pathogenic microbes or proteins. However, these strategies have their limitations, such as weakened viral-induced immunogenicity and incomplete inactivation. For the prevention and treatment of pericardial effusion syndrome, the recombinant subunit vaccine provided better prevention and control of the disease compared to a commercial inactivated vaccine available in the market (Shah et al., 2012). Moreover, the production of subunit vaccines has a number of advantages, that avoid the disadvantages which can avoid the disadvantages of inactivated vaccines, of inactivated vaccines such as the cost of antigen production, unattenuated virulence and incomplete inactivation. Therefore, recombinant protein subunit vaccines are expected to be more ideal as new vaccines after attenuated live vaccines and inactivated vaccines.

Within the capsid structure of the adenovirus, the hexon, penton and fiber proteins contain adenovirus-specific antigenic components, and the epitope located on the hexon protein is the standard antigen used for diagnosis of different serotypes. Generally, the antigen components of mammalian adenoviruses are most sensitive to the pressure of immune selection. Therefore, in this study, the part of the human adenovirus type 7 that spans the hexon was selected as the antigen to develop the subunit vaccine. The recombinant subunit vaccine was constructed using the Bac-to-Bac insect baculovirus expression system.

The antigen, after stimulating DCs, promoted DCs maturation and the antigenic peptide-MHC molecular complex was presented to naive T cells (Iwasaki and Medzhitov, 2015). During antigen presentation, DCs not only up-regulated the expression of MHC II, CD80 and CD86 to activate T cells, but also released a variety of cytokines that affected the direction of T cells differentiation (Moser and Murphy, 2000). For example, IL-12 and IFN-γ promoted the differentiation of primary CD4^+^T cells into Th1 cells and mediated T-cell-assisted immune responses (Napolitani et al., 2005). Other cytokines, such as TNFα and IL-6, play an important role in the regulation of other immune cells (Moussion and Girard, 2011). Consistent with previous studies, the rBV-hexon was able to promote DC maturation *in vitro* and induce DCs to secrete a variety of cytokines, which enhanced antiviral and immunomodulatory activities.

As mentioned above, we noted that the expression levels of the rBV-hexon-activated DCs surface markers were similar to those for LPS-activated DCs. The LPS receptor TLR4 can recognize the viral glycoprotein (Zhang et al., 2003) and LPS-induced cytokines are mainly related to NF-κB, downstream of the TRL4-induced pathway (Kawai and Akira, 2011). As NF-κB can activate many pro-inflammatory cytokines including TNFα and IL-6 (Kawai and Akira, 2011), we speculated that the TLR4/NF-κB pathway might be involved in the activation of DCs by rBV-hexon. As expected, the addition of the NF-κB inhibitor significantly reduced DCs surface marker expression, whereas while after the treatment of TLR4 inhibitor, only the number of MHC II- and CD86-positive cells was significantly reduced after treatment with the TLR4 inhibitor. In terms of cytokines, the two inhibitors significantly inhibited the secretion of TNFα, IFNγ and IL-6. This indicated that TLR4 and NF-κB were involved in rBV-hexon-activated DC maturation.

In subsequent *in vivo* experiments in mice, we found that rBV-hexon significantly increased the titer of mouse serum anti-adenovirus type 7 specific antibodies and neutralizing antibodies, and promoted T-lymphocyte activation. In addition, pathological examination of tissues harvested from immunized mice showed that rBV-hexon did not induce any significant damage to the muscles of various important organs and around the injection site. Taken together, these factors propose the safety and high immunogenicity of the subunit vaccine. A limitation of this study is that we did not perform experiments on protection against virus infection. We will establish an adenovirus type 7 working virus model in further studies to verify the protective effect of rBV-hexon-induced antibodies.

In conclusion, the recombinant subunit vaccine rBV-hexon constructed in this study not only stimulated the maturation of DCs, but also produced high titers of adenovirus specific antibodies and neutralizing antibodies *in vivo*, and promoted the activation of T lymphocytes. These results provide experimental basis for future studies into the development and validation of an adenovirus type 7 subunit vaccine in future.

## Data availability statement

The raw data supporting the conclusions of this article will be made available by the authors, without undue reservation.

## Ethics statement

The animal study was reviewed and approved by Institutional Animal Care and Use Committee (IACUC) of the Changchun University of Chinese Medicine.

## Author contributions

Conceived and designed the experiments: YQL, JH, JF and YZ. Performed the experiments: YRL, XY, RZ, ZX, SL, YL, GS and CG. Analyzed the data: RZ, JF and JH. Contributed reagents/materials/analysis tools: YZ, RZ, ZX, SL, YL, GS and CG. Wrote the paper: YRL, XY and YQL. All authors contributed to the article and approved the submitted version.
