# Supplementary material for: Human adenovirus type 7 subunit vaccine induces dendritic cell maturation through the TLR4/NF-κB pathway is highly immunogenic
Source: Front Cell Infect Microbiol. 2023 Apr 12;13:1117230. doi: 10.3389/fcimb.2023.1117230 (PMC10130362; doi:10.3389/fcimb.2023.1117230)
Supplement: Supplementary file 1 [file DataSheet_1.pdf]

Supplementary figures

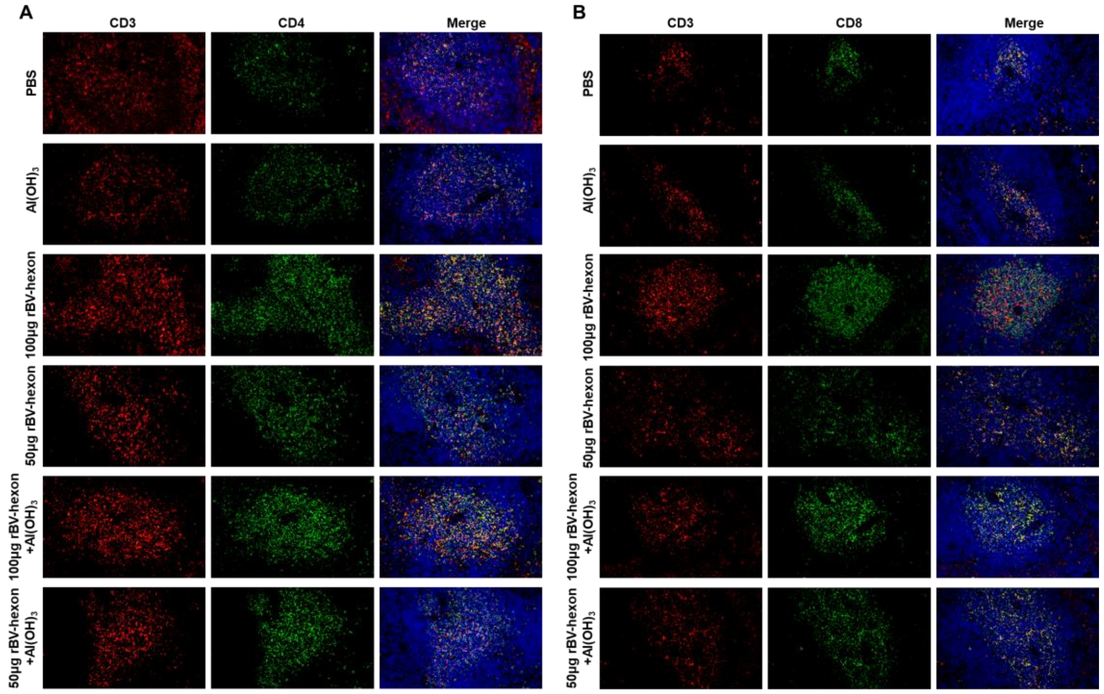

Figure S1. The ratio of CD3+CD4+ and CD3+CD8+ cells in spleen lymphocytes was detected by fluorescence assay.

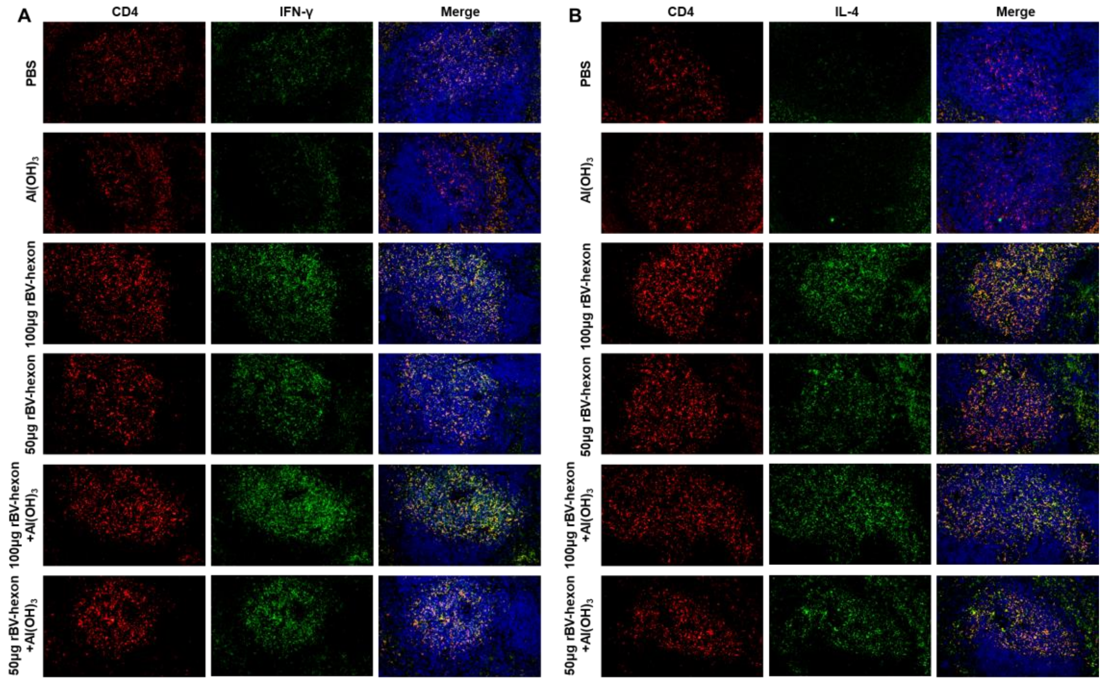

Figure S2. Immunofluorescence assay was used to detect the secretion of IFN-γ and IL-4 to evaluate the activation of CD4+T-cell.

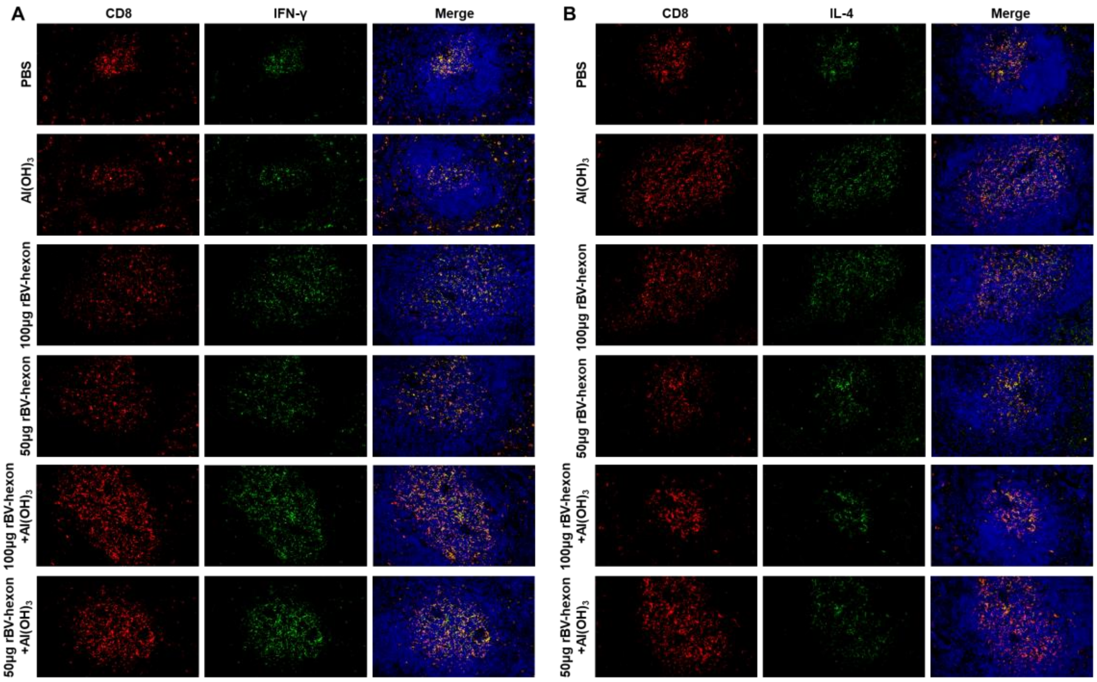

Figure S3. Immunofluorescence assay was used to detect the secretion of IFN-γ and IL-4 to evaluate the activation of CD8+T-cell.
